# Impact of periodic health examination on surgical treatment for uterine fibroids in Beijing: a case-control study

**DOI:** 10.1186/1472-6963-10-329

**Published:** 2010-12-07

**Authors:** Hai-Yun Wu, Ling-Ling Yang, Shan Zhou

**Affiliations:** 1 Institute of Geriatric Cardiology, Chinese PLA General Hospital, 28 Fuxing Road, Beijing 100853, PR China; 2 Department of Gynecology & Obstetrics, Chinese PLA General Hospital, 28 Fuxing Road, Beijing 100853, PR China; 3 Chinese Navy General Hospital, 6 Fucheng, Beijing 100048, PR China

## Abstract

**Background:**

During the past 2 decades, there has been a rapid proliferation of "health examination center (HEC)" across China. The effects of their services on public's health have not been systemically investigated. This study aimed to assess the impact of periodic health examination (PHE) at HEC on surgical treatment for uterine fibroids in Beijing residents.

**Methods:**

We identified 224 patients with a primary diagnosis of uterine fibroids who had surgical treatment at four Level-1 general hospitals in Beijing, from June 1, 2009 to October 20, 2009. Controls were women who did not have surgery for uterine fibroids, matched (1:1 ratio) for age (within 2 years). A standard questionnaire was used to inquire about whether participants had PHE at HEC during the previous 2 years.

**Results:**

PHE at HEC within 2 years were associated with surgical treatment for uterine fibroids. Odds ratios was 4.05 (95% CI, 2.61-6.29 P < 0.001), after adjustment for marital status, whether have children, annual family income, health insurance, education level and self-rated uterine fibroids-related symptom severity.

**Conclusions:**

Our study showed PHE currently provided at HEC in China were associated with significantly increased use of surgical treatment for uterine fibroids in women. Further studies are needed to assess the effects of PHE on clinical as well as on broad societal outcomes in Chinese in contemporary medical settings.

## Background

An annual periodic health examination (or preventive health examination, PHE) used to be a privilege for government workers in China. However, with China's transition to a market economy, the nation's health care has also transformed to a predominantly fee-for-service system [1,2]. Since the 1980 s, particularly during the 1990 s, there has been a rapid proliferation of health examination centers (HEC) across China, especially in large cities. The majority of these centers, which presently total nearly 5000, are set up by public hospitals, while a growing number are created at private institutions [3]. More and more state-owned or private enterprises and government institutes are paying for annual PHE for their employees. Consumers usually refer themselves to these HEC without physician referral. The examination content varies from simple physical examination, chest X-ray, electrocardiogram, abdomen ultrasound and urine and blood tests to whole body CT or MRI imaging and a battery of hundreds of biochemical laboratory studies, with costs ranging from less than 100 RMB (≈$15) to more than 10,000 RMB(≈$1500). For female consumers, gynecological ultrasound examinations are generally included in the health examination program. To date, no governmental regulations or professional guidelines for HEC have been implemented in China, and the impact of their services on public health has not been systemically investigated.

Uterine fibroids are the most common type of tumor in the female reproductive system and leading cause of hysterectomy. A population-based study in the United States found a cumulative incidence of uterine fibroids of greater than 66% by ultrasound examination of women approaching age 50 years [4,5]. A recent statistic from the Chinese Society of Health Management for nealy 500,000 adult Chinese women who had health examinations during the year of 2008 showed a prevalence of ultrasound diagnosed uterine fibroids of 14.7% (unpublished data). Uterine fibroids may cause considerable symptoms, including menorrhagia, dysmenorrheal and pelvic pain and pressure in some patients, necessitating surgical intervention [4,5]. Treatment for uterine fibroids, however, is generally indicated only when symptoms are present that are severe enough to be unacceptable to the patient. There is no evidence that women with no symptoms or with mild symptoms benefit from intervention [6]. Significant differences of hysterectomy rates for uterine fibroids between countries [7] and among ethnic groups [8] have been reported in numerous studies, raising questions of overused or underused medical care from medical profession [7,9,10] and from the lay public [11], implicating high subjectivity in decision making.

During the past decades, we observed a rapidly growing number of women who underwent hysterectomy for uterine fibroids in China. Economic development, easier access to health care, improved surgical skill of the gynecologists might all contribute to this but other socioeconomic factors, especially the marketization of China's healthcare system might also play important roles. We conducted this case-control study to assess the impact of PHE performed at HEC on surgical treatment of uterine fibroids in Beijing, China.

## Methods

### Selection of cases and controls

Hospitals in China are officially classified into 3 levels: level-1, level-2 and level-3. Level-1 and level-2 hospitals are further classified into first rank, second rank, and third rank, with level-1 first rank hospitals as the most selective and top-quality category. Currently, there are 48 level-1 and 74 level-2 hospitals in the urban area of Beijing. Because all level-3 and most of the level-2 hospitals have no gynecology and obstetrics inpatient departments, we randomly chose 2 first rank and 2 second rank general hospitals using a random number table, among the 48 level-1 hospitals, to select study subjects.

Consecutive patients admitted to these four hospitals with a primary diagnosis of uterine fibroids were identified through the hospital's computerized information system. Patients were eligible to be included if they fulfilled these criteria: 1) age 20-50 years; 2) resident of the urban area of Beijing; 3) no history of major chronic disease (cardiovascular, pulmonary or renal diseases, diabetes, uncontrolled hypertension, asthma, allergies, and neoplasm); and 4) undergoing surgical treatment, either hysterectomy or myomectomy, for uterine fibroids. Patients with corrected or added diagnosis after surgery were not excluded. From June 1, 2009 to October 20, 2009, a total of 253 eligible patients were identified, and 29 (mean age, 44.6 years) declined to participate (22 due to not in Beijing, 7 due to other reasons), leaving 224 in the study group.

**Figure 1:**
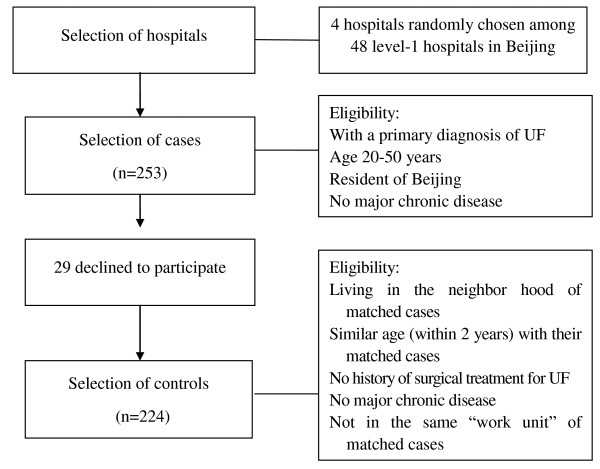
**Flow chart of selecting study subjects**. UF: uterine fibroids.

Controls were selected at random from women who were living in the same apartment building or neighborhood of the patients, without history of major chronic diseases, without previous hysterectomy, in the same age group (within 2 years), and matched 1:1 for each case. Because PHE is often organized by work unit (institute or enterprise) and because women at the same work unit often seek medical care from the same hospital, women from the same 'work unit' of the matched case were not eligible for selection as a control. (Figure 1).

All participants provided written informed consent and the study was approved by the Chinese PLA General Hospital Institutional Review Board.

### Questionnaire survey

We used a standardized questionnaire to collect data from all cases and controls, including age, marital status, whether they had at least one child, employment status, family income, health insurance, education, whether they had been diagnosed with uterine fibroids, and, if so, the first year they were told the diagnosis, self-rated uterine fibroids related symptom severity, and whether they had PHE during the previous 2 years (Additional file 1). The questionnaire was completed mostly by participants at home, and in few cases, by telephone.

Health insurance was categorized into mainstream insurance (including urban employee basic health insurance scheme, Government Insurance Scheme and Labor Insurance Scheme) and other insurance (including other non-commercial or commercial insurance) [12]. Self-reported diagnosis of uterine fibroids included those made by a physician or by the HEC. Self-rated uterine fibroids related symptom severity was the mean score of uterine fibroids related symptoms the participants experienced during the 6 months prior to the date of surgical treatment for uterine fibroids; for controls, the surgical date of matching case was used at the index date. Symptoms of concern included menorrhagia, dysmenorrheal and pelvic pressure, each being scored on a scale of 0-5, with 0 being without symptom and 5 being very severe. These rating were condensed into 3 categories: mild (<2), moderate (2-4) and severe (>4). Because the etiology of uterine fibroids remains poorly understood and no risk factors have been firmly established, the questionnaire did not address possible risk factors, such as age at menarche, parity and first birth, years since last birth, and family history [13,14].

### Definition of PHE

For this study, we defined PHE as self-referred or institute organized health examination at a HEC, whether located in Beijing or in any other place. To avoid bias, the examination did not necessary include gynecological or pelvic ultrasound examination since examinees with uterine fibroid related symptoms might be more likely to ask for gynecological and pelvic ultrasound examination. Examinations referred by a clinician after subjects had sought medical care were not considered to be PHE.

### Statistical analysis

For sample size, assuming an odds ratio of 2.0 for surgical treatment for uterine fibroids associated with exposure to at least one health examination in the previous 2 years, 172 cases and 172 matched controls would detect a significant effect with 80% power at the 5% level with a two-sided test, determined by a standard methods for calculating sample size for matched case-control studies [15]. We therefore aimed for at least 220 cases and 220 controls as a fail-safe measure.

Data were entered using Microsoft Excel. Statistical analyses were performed by using the Chinese High Intellectualized Statistical Software (CHISS, Yuanyitang Science & Technology, Beijing, China) and SAS, version 9.1 (SAS Institute Inc., Cary, NC). Results were presented as frequencies for categorical variables and mean ± standard deviation (SD) for continuous variables. Conditional logistic regression was used to calculate unadjusted and adjusted odds ratios (OR) and 95% confidence intervals (95% CI), with surgical treatment for uterine fibroids as the dependent variable, and PHE, level of education, family income, whether they had children, health insurance status, and self-rated uterine fibroids related symptom severity as independent variables. P-values of less than 0.05 were regarded as significant.

## Results

### Characteristics of cases and controls

Table 1 summarizes the characteristics of the 224 cases and 224 controls. There were no differences between cases and controls in terms of age, marital status, family income and level of education. Compared to cases, controls were more likely to have had no children, to be unemployed, and to have no health insurance. Twenty-two (9.8%) participants of the control group reported a previous diagnosis of uterine fibroids, with the majority of those (63.6%) being diagnosed 1 year before the index date. The mean self-rated uterine fibroids related symptom score in the case group was 1.8 ± 1.6, which was significantly higher than that in the control group (1.4 ± 1.2, p = 0.003), and more subjects in the case group reported moderate or severe uterine fibroids related symptom than those in the control group (39.3% vs 25%, χ^2 ^= 10.5, p = 0.001). However, about 60% of case subjects reported mild uterine fibroids related symptoms during the 6 months before their surgical treatment for uterine fibroids.

**Table 1:** Characteristics of cases and controls

	Cases (n = 224)	Controls (n = 224)
**Age (years), mean (SD)**	42.2 (6.2)	42.4 (6.2)
**Marital status**		
Living alone	24 (10.7)	30 (13.4)
Living with spouse/partner	176 (78.6)	168 (75)
Other	24 (10.7)	26 (11.6)
**Children**		
Having at least 1 child	192 (85.7)	172 (76.8)
Having no child	32 (14.3)	52 (23.2)
**Employment status**		
Full-time/part-time work	166 (74.1)	142 (63.4)
Unemployed	36 (16.1)	58 (25.9)
Retired	22 (9.8)	24 (10.7)
**Family income (RMB per year)**		
<50,000	28 (12.5)	36 (16.1)
50,000-100,000	112 (50.0)	124 (55.4)
>100,000	84 (37.5)	64 (28.6)
**Health insurance**		
Mainstream insurance	138 (61.6)	102 (45.5)
Other insurance	12 (5.4)	8 (3.6)
No insurance	74 (33.0)	114 (50.9)
**Education**		
≤high school	78 (34.8)	98 (43.8)
College	100 (44.6)	95 (42.4)
Graduate school	46 (20.5)	31 (13.8)
**Previous diagnosis of uterine fibroids**		
Within 1 year	160 (71.4)	8 (3.6)
1 year before	64 (28.6)	14 (6.3)
No diagnosis	0 (0)	202 (90.2)
**Self-rated uterine fibroids related symptom score, mean (SD)**	1.8 (1.6)	1.4 (1.2)
Mild	136 (60.7)	168 (75.0)
Moderate	62 (27.7)	43 (19.2)
Severe	26 (11.6)	13 (5.8)
**Annual health examination**		
Having at least 1 health examination	138 (61.6)	62 (27.7)
Not having health examination	86 (38.4)	162 (72.3)

Data are number (%), unless otherwise stated; 'Other' in marital status reflects living with other family members; 'Unemployed' includes homemaker; mainstream insurance includes urban employee basic health insurance scheme, Government Insurance Scheme and Labor Insurance Scheme. Percentages may not total 100% because of rounding.

### Impact of PHE on surgical treatment of uterine fibroids

As shown in Table 1 within the 2 years prior to the date of their surgical treatment for uterine fibroids, 138 of the 224 (61.6%) case subjects had at least 1 health examination at HEC, while only 62 (27.7%) of their matched controls had this examination during the same periods, corresponding to an unadjusted, matched OR of 4.19 (95% CI, 2.84 ~ 6.19, p < 0.01). After adjusting for marital status, whether they had children, annual family income, health insurance, education level and self-rated uterine fibroids-related symptom severity, the multivariable OR for surgical treatment associated with health examination remained quantitatively and statistically significant at 4.05 (95% CI, 2.61-6.29 p < 0.001) (Table 2).

**Table 2:** Odds ratios for surgical treatment for uterine fibroids by annual health examination and other variables

	Unadjusted OR (95% CI)	Adjusted OR (95% CI) *
**Marital status**		
Living alone	1.0 (Ref)	1.0 (Ref)
Living with spouse/partner	1.31(0.74-2.33)	1.03(0.55-1.95)
Other	1.15 (0.53-2.50)	0.47 (0.27-1.70)
**Children**		
Having at least 1 child	1.0 (Ref)	1.0 (Ref)
Having no child	0.55 (0.89-0.34)	0.46 (0.27-0.80)†
**Employment status**		
Full-time/part-time work	1.0 (Ref)	1.0 (Ref)
Unemployed	0.53 (0.85-0.33)	0.52(0.26-1.06)
Retired	0.78 (1.46-0.42)	0.87 (0.39-1.89)
**Family income (RMB per year)**		
<50,000	1.0 (Ref)	1.0 (Ref)
50,000-100,000	1.16 (0.67-2.02)	1.07(0.68-1.70)
>100,000	1.69 (0.94-3.04)	1.37(0.71-2.67)
**Health insurance**		
Mainstream insurance	1.0 (Ref)	1.0 (Ref)
Other insurance	1.11 (0.44-2.81)	0.55(0.20-1.51)
No insurance	0.48 (0.71-0.33)	0.62(0.40-0.96)‡
**Education**		
≤High school	1.0 (Ref)	1.0 (Ref)
College	1.32 (0.88-1.99)	1.80(0.98-3.28)
Graduate school	1.86 (1.09-3.20)	1.57(0.87-2.85)
**Self-rated uterine fibroids related symptom score**		
Mild	1.0 (Ref)	1.0 (Ref)
Moderate	1.81 (1.16-2.83)	0.94(0.40-2.20)
Severe	2.51 (1.26-4.98)	1.51(0.69-3.30)
**Annual health examination**		
Not having health examination	1.0 (Ref)	1.0 (Ref)
Having at least 1 health examination	4.19 (2.84-6.19)	4.05(2.61-6.29)§

Categories of variables explanations are as in Table 1. OR, odds ratio; CI, confidence interval; Ref, reference category. *Adjusted for variables listed in table. †P = 0.005; ‡P = 0.03; §P = 0.00.

### Effect of other social-economical and clinical factors

Multivariable regression showed that whether they had children (no children versus. at least one child, OR, 0.46, 95% CI, 0.80-0.27, p = 0.005) and health insurance status (no insurance vs. mainstream insurance, OR, 0.62, 95% CI, 0.40-0.96, p = 0.03) were associated with surgical treatment for uterine fibroids. The unadjusted OR associated with moderate and severe uterine fibroids-related symptom severity was 1.81 (moderate *vs*. mild, 95% CI, 1.16-2.83) and 2.51 (severe vs. mild, 95% CI, 1.26-4.98), respectively. However, after adjustment for other variables, the OR became 0.94 (95% CI, 0.40-2.20, p = 0.89) and 1.51(95% CI, 0.69-3.30, p = 0.30), respectively. We did not find a statistically significant association between marital status, family income, or education level and surgical treatment for uterine fibroids. (Table 2)

## Discussion

Since the conception of PHE--the medical evaluation of ostensibly healthy adults by physicians at regular intervals, great shifts have occurred both in the content of examination and in the legitimacy with which this examination has been viewed [16]. Several studies have shown that receipt of PHE is associated with increased use of evidence-based preventive care and cancer screening [17,18]. PHE are also believed to strengthen physician-patient relationships [19-21]. However, concerns PHEs are an inefficient use of physicians' time and involve unnecessary laboratory testing have been raised by some observers in western countries where PHE has been a fundamental part of medical practice for decades [22,23].

In this case-control study of Beijing residents, we found that more than 60% of subjects who underwent surgical treatment for uterine fibroids, had at least 1 PHE at HEC during the 2 years prior to their surgery, which was much higher than that of their matched controls (27.7%), and multivariable analysis showed a statistically significant association between PHE and surgical treatment for uterine fibroids, even after adjustment for uterine fibroids-related symptom severity and other social-economic factors. Whether surgical treatment is overused in those who received PHE or underused in those who did not receive PHE, or both, remain to be elucidated.

Patient utilization of healthcare is affected by various environmental and provider-related factors [24]. Powell et al. observed that, in the United States, underserved ethnic minority women had elevated rates of past hysterectomy for benign conditions that could not be accounted for by known risk factors, and concluded that disparity in the form of overuse in these disadvantaged groups might exist [8]. Geller and associates found that in hysterectomy decision-making, non-clinical factors played a smaller, secondary, although statistically significant role compared to that of clinical and patient factors [25]. However, non-clinical factors may play greater roles in contemporary China, where health literacy remains relatively low, with only 6.48% of residents having adequate health literacy [26], and hospital and physician behavior is largely profit driven without a proper system to monitor and assess outcomes [1,2,27]. Furthermore, HECs in China, which are promoting their service vigorously and directly to the public, may tend to highlight the importance of "abnormal findings" in their clients.

An alternative explanation is that surgical treatment for uterine fibroids might be underused in those women who did not have health examinations.

Although the present study was not aimed to assess the appropriateness of surgery for uterine fibroids, we found that about 60% of women who had underwent surgical treatment reported mild uterine fibroids related symptom during the 6-month prior to their surgery, but there was no significant association between symptom severity and surgical treatment by multivariable regression. These findings suggested that overuse of surgical treatment in these patients was highly possible.

Previous studies of western populations reported that underserved subgroups might be at particular risk for elective hysterectomy, and women of lower education had higher rates of hysterectomy for benign conditions than women of higher education [8]. In the present study, however, we found no association between family income or education level and surgical treatment for uterine fibroids in Beijing residents. We were unable to explain the different results because other confounding factors, such as access to health care, were not addressed in this study. As expected, we found that women without children or without medical insurance were less likely to seek surgical treatment for uterine fibroids.

Several limitations of the present study should be mentioned. Ideally, a large-scale, randomized, prospective trial would provide more convincing evidence on the effects of PHE on population health, including their impact on healthcare utilization and demand. However, the trial would be costly and difficult or even impossible to perform. Data for this study were from survey that relied on self-report of key variables, which could not rule out reporting bias completely. For example, the controls, especially those without a diagnosis of uterine fibroids, would tend to under-report their symptoms compared to the cases, but this would attenuate the odds of surgical treatment associate with PHE. Another limitation of our study was that non-surgical treatment for uterine fibroids, such as medical therapy and uterine fibroid embolization [6], were not included. However, medical therapy for uterine fibroids, except herbal medicine recommended by traditional Chinese medicine practitioners, is seldom used in China, and uterine fibroid embolization is currently only performed by a few medical centers; therefore, we think this limitation would not considerably weaken the primary objective of this study.

## Conclusions

In conclusion, our study showed that the PHE currently performed at HEC in China, were associated with significantly increased use of surgical treatment for uterine fibroids in women, providing further evidence that nonclinical factors may play important roles in women's decision to undergo hysterectomy for benign conditions, particularly in a fee-for-service healthcare system. Our findings also suggested that surgical treatment for uterine fibroids might be overused in those patients who had PHE within 2 years. Further studies are needed to assess the effects of PHE on clinical as well as on broad societal outcomes in Chinese in contemporary medical settings.

## Abbreviations

PHE: periodic health examination; HEC: health examination center.

## Competing interests

The authors declare that they have no competing interests.

## Authors' contributions

HYW designed the study, developed the questionnaire and drafted the manuscript. LLY participated in design of the study and development of the questionnaire, data collection and data entry. SZ participated in data collection and checkup. All authors participated in discussion, revision and approved of the final manuscript.

## Pre-publication history

The pre-publication history for this paper can be accessed here:

http://www.biomedcentral.com/1472-6963/10/329/prepub

## Supplementary Material

Additional file 1 **Uterine fibroids related symptoms questionnaire**. This standardized questionnaire was used to collect data from all cases and controls, including age, marital status, whether they had at least one child, employment status, family income, health insurance, education, whether they had been diagnosed with uterine fibroids, and, if so, the first year they were told the diagnosis, self-rated uterine fibroids related symptom severity, and whether they had PHE during the previous 2 years. Click here for file

## References

[B1] Blumenthal D Hsiao W Privatization and its discontents--the evolving Chinese health care system N Engl J Med 2005 353 1165 1170 10.1056/NEJMhpr051133 16162889

[B2] Ho CS Gostin LO The social face of economic growth: China's health system in transition JAMA 2009 301 1809 1811 10.1001/jama.2009.555 19417198

[B3] Huang JS Wang Y Zhang L Report on current status of health management related facilities in China 2009 Beijing: Peking Union Medical College Press

[B4] Van Voorhis B A 41-year-old woman with menorrhagia, anemia, and fibroids: review of treatment of uterine fibroids JAMA 2009 301 82 93 10.1001/jama.2008.791 19050179

[B5] Day Baird D Dunson DB Hill MC Cousins D Schectman JM High cumulative incidence of uterine leiomyoma in black and white women: ultrasound evidence Am J Obstet Gynecol 2003 188 100 107 10.1067/mob.2003.99 12548202

[B6] Goodwin SC Spies JB Uterine fibroid embolization N Engl J Med 2009 361 690 697 10.1056/NEJMct0806942 19675331

[B7] Coulter A McPherson K Vessey M Do British women undergo too many or too few hysterectomies? Soc Sci Med 1988 27 987 994 10.1016/0277-9536(88)90289-4 3067370

[B8] Powell LH Meyer P Weiss G Matthews KA Santoro N Randolph JF Jr Schocken M Skurnick J Ory MG Sutton-Tyrrell K Ethnic differences in past hysterectomy for benign conditions Women's Health Issues 2005 15 179 186 10.1016/j.whi.2005.05.002 16051109

[B9] Jacobson GF Shaber RE Hung YY Changes in rates of hysterectomy and uterine conserving procedures for treatment of uterine leiomyoma Am J of Obstet and Gynecol 2007 196 601 e1-601 10.1016/j.ajog.2007.03.009 17547914

[B10] Broder MS Kanouse DE Mittman BS Bernstein SJ The appropriateness of recommendations for hysterectomy Obstet Gynecol 2000 95 199 205 10.1016/S0029-7844(99)00519-0 10674580

[B11] Masters C Are hysterectomies too common? TIME Magazine http://www.time.com/time/health/article/0,8599,1644050,00.html?cnn=yes Accessed October 27, 2009

[B12] Xu L Wang Y Collins CD Tang S Urban health insurance reform and coverage in China using data from National Health Services Surveys in 1998 and 2003 BMC Health Serv Res 2007 7 37 10.1186/1472-6963-7-37 17335584 PMC1828155

[B13] Wise LA Palmer JR Harlow BL Spiegelman D Stewart EA Adams-Campbell LL Rosenberg L Reproductive factors, hormonal contraception, and risk of uterine leiomyomata in African-American women: a prospective study Am J Epidemiol 2004 159 113 123 10.1093/aje/kwh016 14718211 PMC1847588

[B14] Van Voorhis BJ Romitti PA Jones MP Family history as a risk factor for development of uterine leiomyomas: results of a pilot study J Reprod Med 2002 47 663 669 12216434

[B15] McKeown-Eyssen GE Thomas DC Sample size determination in case-control studies: the influence of the distribution of exposure J Chronic Dis 1985 38 559 568 10.1016/0021-9681(85)90044-X 4008598

[B16] Han PK Historical changes in the objectives of the periodic health examination Ann Intern Med 1997 127 910 917 9382370 10.7326/0003-4819-127-10-199711150-00010

[B17] Boulware LE Marinopoulos S Phillips KA Hwang CW Maynor K Merenstein D Wilson RF Barnes GJ Bass EB Powe NR Daumit GL Systematic review: the value of the periodic health evaluation Ann Intern Med 2007 146 289 300 17310053 10.7326/0003-4819-146-4-200702200-00008

[B18] Fenton JJ Cai Y Weiss NS Elmore JG Pardee RE Reid RJ Baldwin LM Delivery of cancer screening: how important is the preventive health examination? Arch Intern Med 2007 167 580 585 10.1001/archinte.167.6.580 17389289 PMC3443471

[B19] Gordon PR Senf J Is the annual complete physical examination necessary? Arch Intern Med 1999 159 909 910 10.1001/archinte.159.9.909 10326933

[B20] O'Malley PG Greenland P The annual physical: are physicians and patients telling us something? Arch Intern Med 2005 165 1333 1334 15983280 10.1001/archinte.165.12.1333

[B21] Laine C The annual physical examination: needless ritual or necessary routine? Ann Intern Med 2002 136 701 703 11992306 10.7326/0003-4819-136-9-200205070-00013

[B22] Mehrotra A Zaslavsky AM Ayanian JZ Preventive health examinations and preventive gynecological examinations in the United States Arch Intern Med 2007 167 1876 1883 10.1001/archinte.167.17.1876 17893309

[B23] Merenstein D Daumit GL Powe NR Use and costs of nonrecommended tests during routine preventive health exams Am J Prev Med 2006 30 521 527 10.1016/j.amepre.2006.02.003 16704947

[B24] Phillips KA Morrison KR Andersen R Aday L A Understanding the context of healthcare utilization: assessing environmental and provider-related variables in the behavioral model of utilization Health Serv Res 1998 33 3 Pt 1 571 596 9685123 PMC1070277

[B25] Geller SE Burns LR Brailer DJ The impact of nonclinical factors on practice variations: the case of hysterectomies Health Serv Res 1996 30 729 750 8591927 PMC1070090

[B26] Ministry of Health of the Republic of China The overall percentage of Chinese residents with adequate health literacy is 6.48% http://news.xinhuanet.com/health/2009-12/18/content_12666690.htm Accessed November 23, 2009

[B27] Ma J Lu M Quan H From a national, centrally planned health system to a system based on the market: lessons from China Health Aff (Millwood) 2008 27 937 948 10.1377/hlthaff.27.4.937 18607026

