# Supplementary material for: Impact of periodic health examination on surgical treatment for uterine fibroids in Beijing: a case-control study
Source: BMC Health Serv Res. 2010 Dec 7;10:329. doi: 10.1186/1472-6963-10-329 (PMC3002351; doi:10.1186/1472-6963-10-329)
Supplement: Additional file 1 — Uterine fibroids related symptoms questionnaire. This standardized questionnaire was used to collect data from all cases and controls, including age, marital status, whether they had at least one child, employment status, family income, health insurance, education, whether they had been diagnosed with uterine fibroids, and, if so, the first year they were told the diagnosis, self-rated uterine fibroids related symptom severity, and whether they had PHE during the previous 2 years. [file 1472-6963-10-329-S1.DOC]

**Uterine Fibroids Related Symptoms Questionnaire**

| **Name**: | **Date of Birth** _____Year ___Month _____Day | |
| --- | --- | --- |
| **Institute**: | | |
| **Family Address:** | | |
| **Tel**： | | **E-mail：**_______________________________ |

**Education:** □Junior high school or less □Senior high school □University or college □Graduate school or higher

**Marital Status:** □Not married □Married □Divorced □Widowed □Other

**Employment:** □Full time work □Part time work □Retired □Home work □No employment □Other

**Family Income in a Year：**□<50,000 RMB □50,000-100,000 RMB □>100,000 RMB

**Medical Insurance：** □Government Insurance Scheme □Urban Employee Basic Health Insurance Scheme □Labor Insurance Scheme □Commercial Medical Insurance □No Medical Insurance □Other

**Please try your best to remember, and answer the following questions:**

1. During the 2 years prior to the date of________________, have you ever had health examination at the Health Examination Center located in Beijing or in any other places？

□No □Had once □Had twice or more

2. Have you ever told by physicians at hospital or at the Health Examination that you had uterine fibroids?

□Yes □No

3. If you were diagnosed as to have uterine fibroids, when is the first time you were told the diagnosis？

□Within 1 year □1year before

4. During the 6 months prior to the date of________________, have you ever experienced the following symptoms, and if “yes”, what do you think the severity?

A. Menorrhagia

□Never □Very mild □Mild □Moderate □Severe □Very severe

B. Dysmenorrheal

□Never □Very mild □Mild □Moderate □Severe □Very severe

C. Pelvic pressure, whether during the periods or at any other time

□Never □Very mild □Mild □Moderate □Severe □Very severe

**Date:** _____Year ___Month _____Day
